# Mechanism Research of PZD Inhibiting Lung Cancer Cell Proliferation, Invasion, and Migration based on Network Pharmacology

**DOI:** 10.2174/0113816128296328240329032332

**Published:** 2024-04-03

**Authors:** Fan Feng, Ping Hu, Lei Peng, Jun Chen, Xingkui Tao

**Affiliations:** 1 School of Biological and Food Engineering, Suzhou University, Anhui 234000, China;; 2 Anhui Longruntang Biotechnology Co., Ltd, Anhui 234000, China

**Keywords:** Network pharmacology, *Pinellia ternata*, lung cancer, traditional Chinese medicine, PI3K/AKT signaling pathway, KEGG analysis

## Abstract

**Background:**

A classic Chinese medicine decoction, *Pinellia ternata* (Thunb.) Breit.-*Zingiber officinale* Roscoe (Ban-Xia and Sheng-Jiang in Chinese) decoction (PZD), has shown significant therapeutic effects on lung cancer.

**Objective:**

This study aimed to explore and elucidate the mechanism of action of PZD on lung cancer using network pharmacology methods.

**Methods:**

Active compounds were selected according to the ADME parameters recorded in the TCMSP database. Potential pathways related to genes were identified through GO and KEGG analysis. The compound-target network was constructed by using Cytoscape 3.7.1 software, and the core common targets were obtained by protein-protein interaction (PPI) network analysis. Batch molecular docking of small molecule compounds and target proteins was carried out by using the AutoDock Vina program. Different concentrations of PZD water extracts (10, 20, 40, 80, and 160 μg/mL) were used on lung cancer cells. Moreover, MTT and Transwell experiments were conducted to validate the prominent therapeutic effects of PZD on lung cancer cell H1299.

**Results:**

A total of 381 components in PZD were screened, of which 16 were selected as bioactive compounds. The compound-target network consisting of 16 compounds and 79 common core targets was constructed. MTT experiment showed that the PZD extract could inhibit the cell proliferation of NCI-H1299 cells, and the IC_50_ was calculated as 97.34 ± 6.14 μg/mL. Transwell and wound-healing experiments showed that the PZD could significantly decrease cell migration and invasion at concentrations of 80 and 160 μg/mL, respectively. The *in vitro* experiments confirmed that PZD had significant therapeutic effects on lung cancer cells, mainly through the PI3K/AKT signaling pathway.

**Conclusion:**

PZD could inhibit the cell proliferation, migration, and invasion of NCI-H1299 cells partially through the PI3K/AKT signaling pathway. These findings suggested that PZD might be a potential treatment strategy for lung cancer patients.

## 1 INTRODUCTION

Lung cancer is a highly heterogeneous malignant tumor. Its morbidity and mortality rates are highest in China. Its prevalence and fatality rate rank first among malignant cancers in China and worldwide [1]. According to the latest data from GLOBOCAN2020, the incidence and mortality of lung cancer in China account for 37.0% and 39.8% of the world, respectively, showing an upward trend in recent years [2]. Therefore, how to prevent and treat lung cancer is a major challenge for the prevention and treatment of malignant tumors. At present, the effective treatment of lung cancer is mainly the development and use of targeted drugs, but its clinical benefit is still limited. Therefore, the clinical prevention and treatment of lung cancer using the multi-component combination of traditional Chinese medicine have been given serious attention [3-5]. A large number of studies have found that traditional Chinese medicine ingredients can induce cell apoptosis and inhibit cell proliferation, which can alleviate symptoms, inhibit tumor development, prolong survival time, and improve the quality of life of patients [6-8]. Due to the lack of support and statistical limitations of large-scale clinical trials based on the concept of evidence-based medicine, traditional Chinese medicine is often used in the adjuvant treatment of lung cancer patients. Many published reports have shown that the efficacy of TCM combined with chemotherapy in lung cancer patients is obvious [9-11], and several natural compounds in traditional Chinese medicine formulations, such as resveratrol, curcumin, and berberine, have anti-cancer properties of inhibiting the occurrence, proliferation, angiogenesis, and metastasis of lung cancer [12, 13]. Unlike the “one drug, one target” approach in Western medicine, TCM theory emphasizes the systematic concept of the entire human body. Due to the complexity of its composition, traditional pharmacological methods that determine its unique mechanism of action through experiments may not be suitable for traditional Chinese medicine research [14-16]. With the rapid development of bioinformatics, emerging network pharmacology based on large databases has become a useful tool for the detailed characterization of complex drug system mechanisms from molecular to pathway levels [17-19]. Network pharmacology conforms to the core philosophy of traditional Chinese medicine as a whole. As a state-of-the-art technology, this method updates the research paradigm from the current “one target, one drug” model to a new “network target, multi-component” model [20-22].

Most lung cancer originates from malignant bronchial mucosal epithelium, and a small part is caused by bronchoalveolar epithelium or glandular lesions. One of the typical symptoms is dyspnea, cough, and expectoration [23-25]. Therefore, traditional Chinese medicine experts in the past paid attention to the use of expectorant products in treatment [26, 27]. PZD recorded in the “Shen Nong's Materia Medica” has a long history of being used in the treatment of cough [28]. It contains only two kinds of tubers of traditional Chinese medicine named Ban-xia (BX) and Sheng-jiang (SJ) in Chinese. CPF mixture (*Coptis chinensis* (Huang-Lian), *Pinellia ternata* (Ban-Xia), and *Fructus trichosanthis* (Tian-Huawen)) containing *Pinellia* could suppress lung cancer cell proliferative switching through transcriptional suppression of FACT and the c-MYC, and *Zingiber officinale* extract was used in combination with many other chemotherapy drugs to treat lung cancer [29-31]. However, the chemical and pharmacological basis of PZD inhibiting human lung cancer has not been comprehensively evaluated using appropriate methods.

In the current research, computational resources and tools were used to investigate the pharmacological network of PZD in lung cancer in order to predict bioactive components and potential protein targets and pathways. Moreover, *in vitro* experiments were also conducted to validate the potential underlying mechanism of PZD in the treatment of lung cancer predicted by network pharmacology. The detailed technical strategy of the current study is shown in Fig. (**1**).

## 2 MATERIALS AND METHODS

### 2.1 Database and Software

A variety of databases and software were used used in this research, including TCMSP database (http://tcmspw.com/tcmsp.php), Gene Cards (https://genecards.org/indexs.shtml), UniProt (http://www.uniprot.org/), String database (https://string-db.org/), David Bioinformatics database (https://david.ncifcrf.gov/summary.jsp), KOBAS (KEGG Orthology Based Annotation System) database (http://kobas.cbi.pku.edu.cn/kobas3), Cytoscape 3.7.1 software, and AutoDock Vina software.

### 2.2 Screening Strategy of Bioactive Components in PZD

All components of the two Chinese medicinal herbs in PZD (Ban-Xia and Sheng-Jiang) were retrieved from the TCMSP database (http://tcmspw.com/) [32, 33]. Oral traditional Chinese medicine must overcome the obstacles in the process of ADME in order to effectively exert its effects. Oral bioavailability (OB) is one of the most significant pharmacokinetic parameters in the ADME process [34]. High OB is usually an important indicator for determining the drug-likeness (DL) index of active substances. Substances with OB ≥ 30% are considered to have high OB. As a qualitative concept applied in drug design to evaluate the medicinal properties of molecules [35], the DL index is useful for the rapid screening of active substances. In the DrugBank database, the average DL index is 0.18. Substances with a DL index ≥ 0.18 are considered to have high medicinal value. As a result, the compounds with DL index ≥ 0.18 and OB ≥ 30% in PZD were selected as bioactive substances, and the database retrieval deadline was October 20^th^, 2023.

### 2.3 Target Protein Prediction of Drug Components in PZD

The protein targets of bioactive substances in PZD were screened from the TCMSP database by using the filter search bar of the related targets of the compound component. Meanwhile, the annotated genome database platform GeneCards, the protein database UniProt, and the online database KOBAS were used to query the human gene name corresponding to the target protein [36].

### 2.4 Constructing a Visual Network of Drugs-bioactive Components-disease Targets

Based on the GeneCards database platform, the relevant target proteins of lung cancer diseases were retrieved. Combined with the identification and screening of drug target proteins in the above step 2.3, lung cancer target proteins were mapped to each other to obtain common target proteins, and then the related information of the drug active ingredient and the common target proteins were imported into Cytoscape 3.7.1 software for data processing. Moreover, the visual network of drugs-bioactive components-disease targets was constructed [37]. Among them, nodes were used to represent key chemical components and disease targets, and solid lines with arrows were used to represent the interaction between nodes.

### 2.5 Construction of Protein-protein Interaction Network and Screening of Core Targets

The String database online was logged in to find the search mode “multiple proteins” box; the common target proteins of PZD and lung cancer were entered into the String database (https://string-db.org/ Version 10.5), and the search species condition was selected as human *(Homo sapiens*). The common target name was converted, the PPI score was set to > 0.7, the visual interaction map of the PPI relationship network was obtained, the free protein that appears outside the network was manually hidden, and the PPI interaction protein relationship map was exported [38]. According to the node degree value, the key core genes of the protein-protein interaction network were screened out.

### 2.6 Gene Ontology and Pathway Enrichment Analysis of PZD Inhibiting Lung Cancer-related Targets

According to the molecular function (MF), cell component (CC), and biological process (BP) functions of the target protein in the cells, GO enrichment was performed to understand the biological processes, such as CC, MF, and BP, in which drug target proteins were mainly participated in. KEGG enrichment analysis was based on the distribution of target proteins in signaling pathway metabolism to further understand the role of the target proteins in cell signal transduction and metabolism pathways. Based on the David Biology database, GO and KEGG pathway enrichment analyses of drugs-key chemical components-disease targets were performed [39]. The target genes were screened according to *p* < 0.05 to obtain *Pinellia ternata*-*Zingiber officinale* target proteins, which were involved in the possible pharmacological regulation mechanism of signal pathways and the main biological processes involved in the regulation. The *p*-value reflected the significant difference in the involvement of the target protein in the regulation of signal pathways. NCBI database was used to search the literature to further screen for other signaling pathways related to lung cancer.

### 2.7 Preparation of PZD Extract

The tubers of *Pinellia ternata* and *Zingiber officinale* in autumn could be dug 12-20 cm deep with a hoe, and then the soil was shaken off to remove the coarse skin and fibrous roots on the surface of the medicinal materials. To prepare the aqueous extract of PZD, 20 g dry tuber of *Pinellia ternata* and 10 g dry tuber of *Zingiber officinale* were ground into fine powder [40, 41], which was purchased from the Bozhou Chinese Herbal Medicine Market and were identified as genuine by associate professor Xingkui Tao of Suzhou University. The powder was soaked in 300 mL of distilled water at room temperature for 12 hours and then boiled for 1 hour. The solution was centrifuged at 10,000 rpm for 30 min, and the supernatant was collected. Before merging the supernatant, the extraction step was repeated twice, and it was then evaporated into dry powder [42]. The dry powder was redissolved in distilled water to a concentration of 10 mg/mL and then filtered with a 0.22 μm pore-size filter. The filtered solution was stored at -20°C for further use.

### 2.8 Cell Culture and Cell Viability Assay

Human lung cancer cells NCI-H1299 were chosen in our lab for the following experiments, which were previously purchased from ATCC cell bank (Shanghai, China). Lung cancer cells were cultured in DMEM medium containing 10% FBS, 100 mg/mL streptomycin, and 100 U/mL penicillin and kept in a humidified chamber containing 5% CO_2_ at 37°C [43].

NCI-H1299 cells were inoculated in 96-well plates with 5,000 cells per well and incubated for 24 hours. After pretreatment with different concentrations of PZD (0, 10, 20, 40, 80, and 160 μg/mL) for 24, 48, and 72 hours, 10 μL of 3-(4,5-dimethylthiazol-2-yl)-2,5-diphenyltetrazolium bromide solution (MTT, 5 mg/ml; Sigma, USA) was added to each well and then cells were cultured at 37°C for another 4 hours. Then, the supernatants were discarded, and 100 μL DMSO was added to each well. The absorbance was measured at 595 nm using an enzyme-linked immunosorbent assay (ELISA) reader [43].

### 2.9 Wound-healing and Transwell Invasion Assay

For the wound-healing assay, NCI-H1299 cells were incubated in 6-well plates with 100% confluence. A denuded area was scrapped using a plastic pipette tip on the cell monolayer. The medium was removed, and the monolayer was washed 3 times with PBS. Then, a medium containing different concentrations of PZD was added to each well, and cell movement entering the wound area was observed under a microscope for 0, 24, and 48 h. As for the Transwell invasion assay experiment, millicell cell culture inserts in 24-well plates were pretreated with 100 μL of cold Matrigel (BD Biosciences, USA, diluted 1:4 with cold PBS) at 37°C for 2 h. Lung cancer cells were seeded to the chamber containing 200 μL of serum-free DMEM with 5,000 cells per well and then incubated with or without PZD at 37°C for 24 h. The invading cells were fixed with 4% paraformaldehyde for 30 min, stained with crystal violet solution for 2 h, and then counted under an optical microscope [44].

### 2.10 Western Blotting

Lung cancer cells were inoculated in 6-well plates (5 × 10^5^ cells/well). After incubation overnight, cells were treated with or without PZD for 24 hours. The cells were harvested using a micro scraper (Corning). The expression levels of GAPDH, PI3K, and AKT were examined by immunoblotting. In short, the whole cell extracts were lysed on ice with RIPA buffer supplemented with phosphatase inhibitor (1 mM NaF and 1 mM Na3VO4) and proteinase inhibitor (0.5% aprotinin, 0.5% leupeptin, and 1% PMSF) for 30 min. Then, the lysates were centrifuged at 14,000 rpm at 4℃ for 10 min. The protein concentration was measured using bovine serum albumin (BSA; Sigma, MO, USA) as the standard. The same amount of protein in each sample was resolved by sodium dodecyl sulfate-polyacrylamide gel electrophoresis (SDS-PAGE) and transferred onto a polyvinylidene fluoride membrane (PVDF, Biorad, USA). Subsequently, the membrane was blocked with 5% BSA at room temperature in Tris-buffered saline-Tween 20 buffer (TBST: 1% Tween 20, 10 mmol/L Tris, 150 mmol/L NaCl, pH 7.4) for 2 h. Then, the blots were incubated with primary antibodies (anti-PI3K, anti-AKT; Abcam, UK) overnight at 4ºC. After washing three times with TBST buffer, the blots were incubated with the secondary antibody (Abcam, UK) at room temperature for 2 h [45, 46]. Immunoreactivity was measured using an advanced ECL assay kit (GE Healthcare, UK) and visualized using a chemiluminescence imaging system.

### 2.11 Statistical Analysis

During the research process, each independent experiment was repeated three times. The statistical analysis between the two groups was conducted by Student's *t-*test using SPSS software (SPSS Inc., USA). Multiple groups of normalized data were analyzed using one-way ANOVA. In statistical analysis, the normality test was applied to each data to determine the statistical test to be used. The data was displayed as mean ± standard deviation. If there were no special conditions required, a *p*-value ≤ 0.05 was usually considered statistically significant.

## 3 RESULTS

### 3.1 Identification of Bioactive Components of PZD and their Target Proteins in Lung Cancer

Through keyword screening, a total of 381 chemical components of *Pinellia ternata*-*Zingiber officinale* were retrieved from the TCMSP database (265 from *Zingiber officinale* and 116 from *Pinellia ternata*). By setting the inclusion criteria to DL ≥ 0.18 and OB ≥ 30%, a total of 16 candidate compounds were retrieved, including 5 from ginger and 13 from *Pinellia*, among which 2 chemical components were found to be common between *Pinellia* and *Zingiber* (Table **1**). Among them, 24-ethylcholest-4-en-3-one, β-sitosterol, poriferast-5-en-3β-ol, cavidine, baicalin, stigmasterol, and other components have drug-like properties of more than 75%, indicating that these chemical components may play a key regulation role in the human body. A total of 150 human target genes were matched from the 16 compound components searched through the TCMSP database. After deduplication, 80 target genes were ultimately obtained.

### 3.2 Construction and Analysis of Visual Network Diagram of Drugs-bioactive Components-disease Targets

There were approximately 22,400 target genes of lung cancer obtained from the GeneCards database by using “lung cancer” as the screening keyword, then a total of 4559 genes were obtained by setting the correlation score greater than or equal to 6. Combining the screened 80 drug targets for mutual mapping, 79 common target genes were obtained. The relationship between these 16 compounds and 79 target proteins was analyzed by Cytoscape software to construct a network visualization map of drugs-key chemical components-disease targets, as shown in Fig. (**2**). A total of 168 edges and 97 nodes were found in the network, as shown in Fig. (**2**). Each candidate compound acted on 9.375 target proteins on average, suggesting that the *Pinellia-Zingiber officinale* pair may exert its effect through multi-component synergistic regulation of multiple targets. The importance of a node was measured in a visual network graph based on its degree value. The higher the value, the greater the likelihood that the node would become a key node. The compounds with relatively high degree values in the network diagram calculated by Cytoscape software were stigmasterol (MOL000449, Degree = 35), β-sitosterol (MOL000358, Degree = 37), carvendine (MOL002670, Degree = 22), baicalein (MOL002714, Degree = 27), and coniferin (MOL000519, Degree = 17), suggesting that these compounds may be the main functional components of PZD.

### 3.3 Construction of PPI Protein Interaction Network and Screening of its Core Targets

Based on the String online database user manual, 79 drugs-key chemical components-disease targets were entered into the multi-protein search box. According to the confidence condition box, a network complexity greater than 0.4 was selected, and the free target protein outside the network diagram was deleted. Then, the visualized PPI protein interaction network diagram was obtained, as shown in Fig. (**3**). In the figure, each target protein was represented by a node, and the edges between the targets represented the interaction relationship. Statistics showed that the network contained a total of 79 nodes and 420 edges, in which an average node degree had a value of 10.6. There were about 39 nodes above the average degree value. The top 10 target nodes, VEGFA, AKT1, TP53, MMP9, FOS, CASP3, and ADAR (1A, 1B, 2A, and 2C), had a degree value greater than 20, suggesting that these may become the key core targets of PZD for the treatment of lung cancer.

### 3.4 GO Enrichment Analysis of Drugs-bioactive Components-disease Targets

**Fig. (3):**
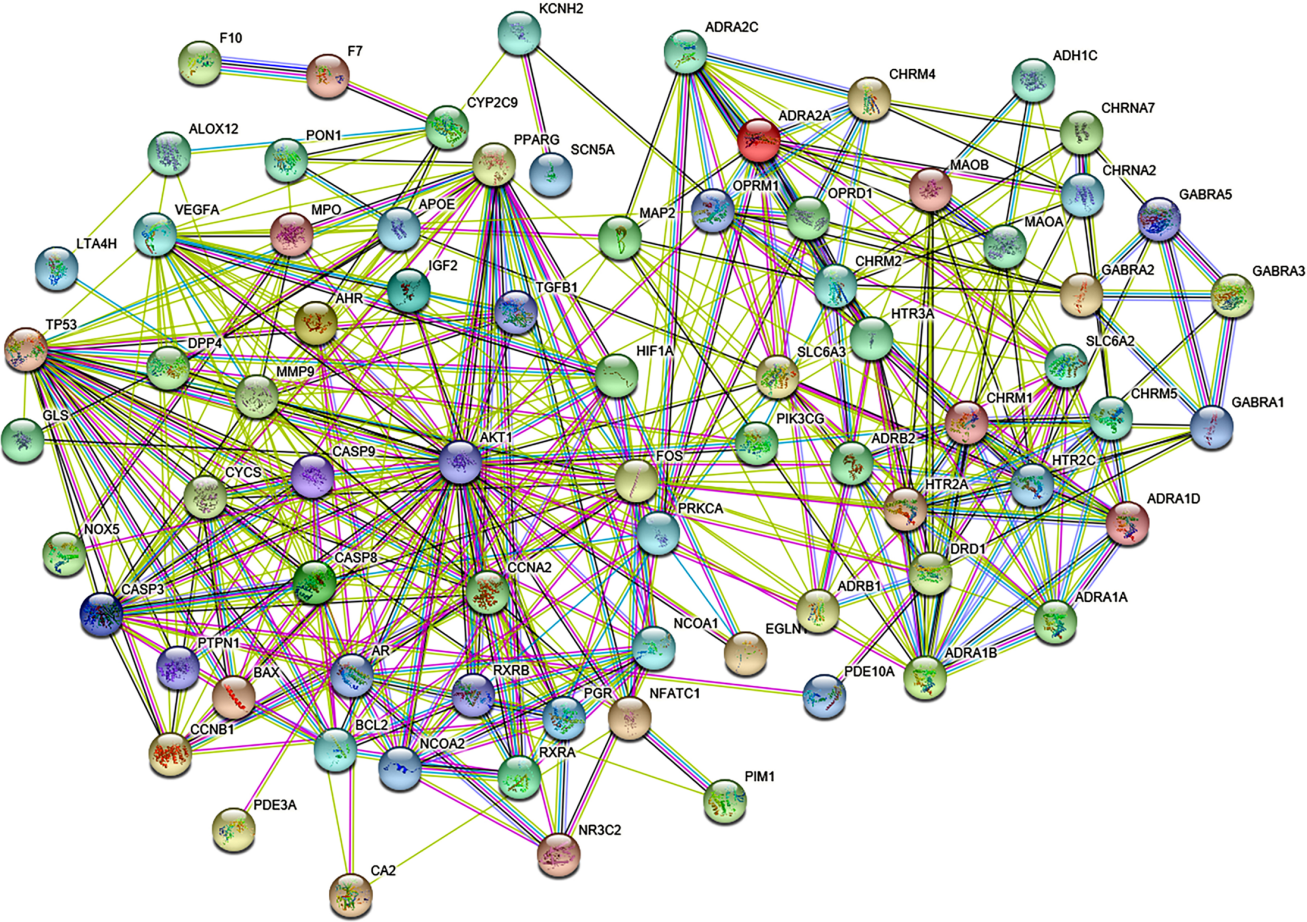
The PPI network of proteins targets of PZD bioactive compounds in lung cancer. The nodes represent protein targets containing protein structure with predicted interaction, where nodes contain structural simulations of individual proteins. The edges represent the interactions between these nodes.

Using the DAVID 6.8 online database, the disease target genes were entered into the list, and the office gene symbol for enrichment analysis was selected for screening. There were about 254 entries of these genes involved in biological process (BP) according to the GO enrichment analysis category, among which the first top 20 ranked biological process items are shown in Fig. (**4**). These GO enrichment items were mainly involved in biological processes, such as drug response, cell signal transduction, positive regulation of cell proliferation, coupled G protein receptor signaling pathway, negative regulation of cell apoptosis process, cell proliferation, cell apoptosis, and aging. At the same time, the enrichment analysis of target genes obtained 37 cell component (CC) entries of these genes. For example, the top 20 cell component entries (Fig. **3**) were mainly involved in cell components, such as plasma membrane, cell nucleus, cytoplasm, cytosol, mitochondria, postsynaptic membrane, cell connections, endoplasmic reticulum, neuron projections, and synapses. Meanwhile, molecular function (MF) was enriched to obtain 68 entries that participate in molecular functions. The top 20 functional molecular entries were mainly involved in protein heterodimer activity (Fig. **3**), protein binding, enzyme binding, drug binding, protein homodimerization activity, zinc ion binding, transcription factor activity, protein kinase binding, ubiquitin protease binding, RNA polymerase II transcription factor activity, and other molecular biological functions.

### 3.5 KEGG Enrichment Analysis of Drugs-bioactive Components-disease Targets

The KEGG enrichment pathway of the target gene was conducted through the DAVID 6.8 database and the KOBAS database. The results showed that a total of 70 KEGG pathways were mainly enriched for drugs-key chemical components-disease targets. KEGG enrichment analysis revealed serotonergic synapses and cGFMP- PKG signaling pathway, adrenaline signaling in cardiomyocytes, calcium signaling pathway, apoptosis, p53 signaling pathway, platinum resistance, non-small cell lung cancer, bladder cancer, renal cell carcinoma, pancreatic cancer, thyroid cancer, intrauterine membrane cancer, and thyroid hormone signaling pathway. As shown in Fig. (**5**), a large number of pathways have been shown to be closely related to tumor regulation and drug resistance pathways, suggesting that the PZD treats lung cancer mainly by regulating tumor cell signaling pathways.

### 3.6 Molecular Docking and Verification of Binding Free Energy **Analysis of the Key Bioactive Component-disease Target**

**Fig. (6):**
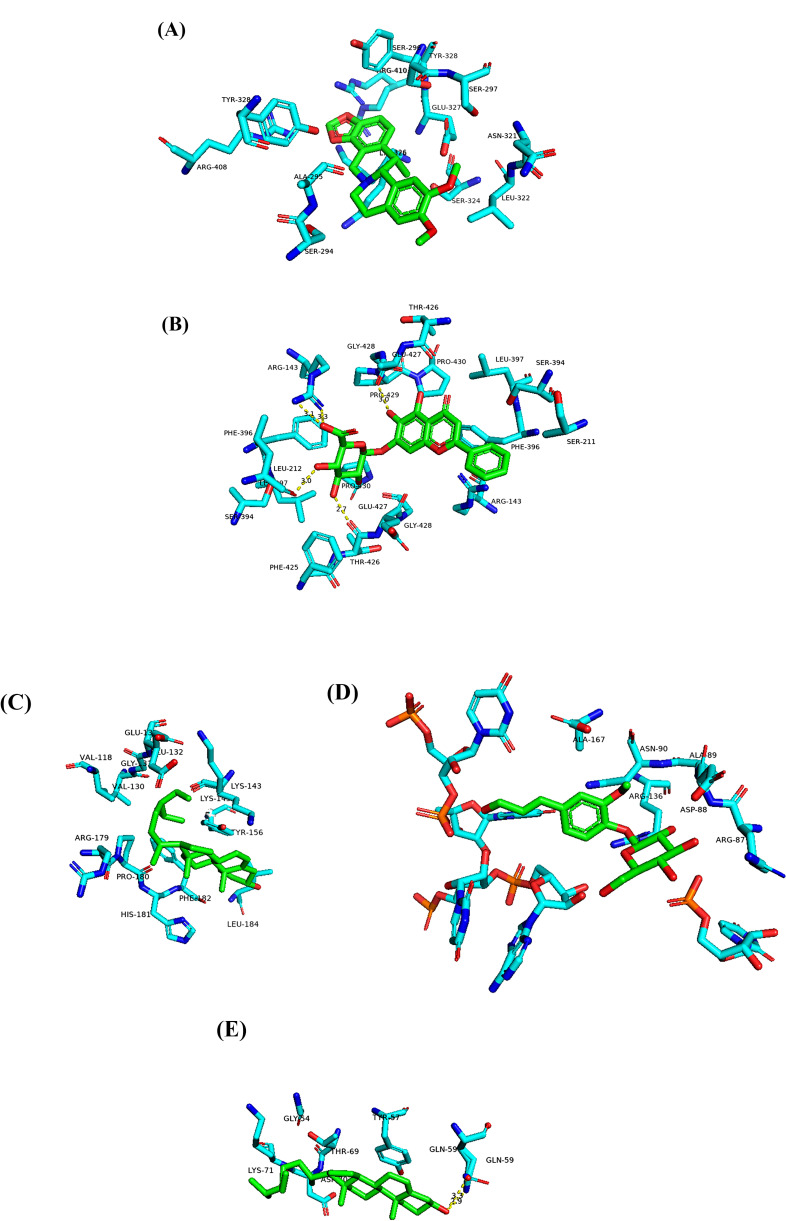
Molecular docking model of key components-protein targets. (**A**) VEGFA-cavidine complex. (**B**) MMP9-baicalin complex. (**C**) AKT1-stigmasterol complex. (**D**) FOS-coniferin complex. (**E**) ADAR1-beta-sitosterol complex.

Based on the AutoDock Vina batch molecular docking program, molecular docking was performed on the five compounds (stigmasterol, β-sitosterol, carvendine, baicalein, and coniferin) with the highest degree in the above network view and the top 5 target proteins (AKT, VEGFA, MMP9, FOS, and ADAR1) in the PPI network graph. The results of the binding free energy analysis are presented in Table **2**, which showed that the effective components of PZD had a strong binding and interaction ability with the target protein (Figs. **6A**-**E**). It was confirmed that PZD could regulate multiple targets of lung cancer with multiple components in the disease pathway mechanism.

### 3.7 PZD Extract Inhibited the Cell Proliferation of NCI-H1299

**Fig. (7):**
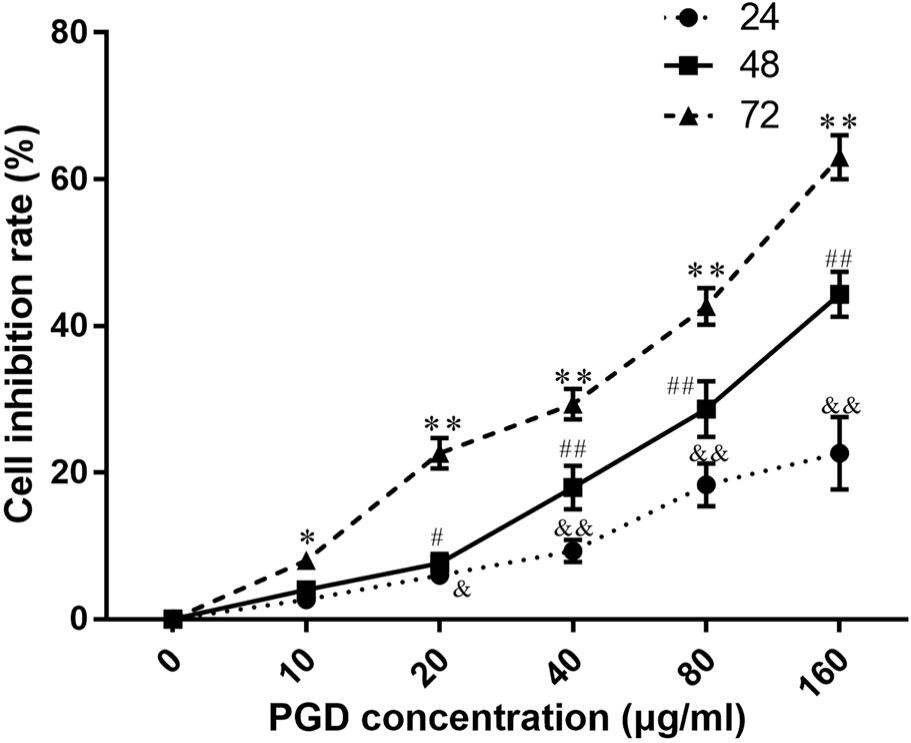
Effects of PZD extracts on the viability of NCI-H1299 cells at different times and concentrations. & and && represent *p* < 0.05 and *p* < 0.01 compared with the 24-hour blank dose group, respectively. # and ## represent *p* < 0.05 and *p* < 0.01 compared with the 48-hour blank dose group, respectively. * and ** represent *p* < 0.05 and *p* < 0.01 compared with the 72-hour blank dose group, respectively.

To verify the antiproliferative effect of PZD on lung cancer cells as postulated from network analysis, NCI-H1299 cells were treated with the water extract of PZD for 24, 48, and 72 hours, respectively. The results showed that the inhibition rate of NCI-H1299 cells increased significantly with the increase in drug concentration and the prolongation of drug action time, indicating that the water extract of PZD had an obvious inhibitory effect on the growth of NCI-H1299 cells. The drug concentration and action time have an interactive effect, and the IC_50_ was calculated as 97.34 ± 6.14 μg/mL at 72 hours (Fig. **7**).

### 3.8 PZD Extract Inhibited the Cell Migration and Invasion of NCI-H1299

**Fig. (8):**
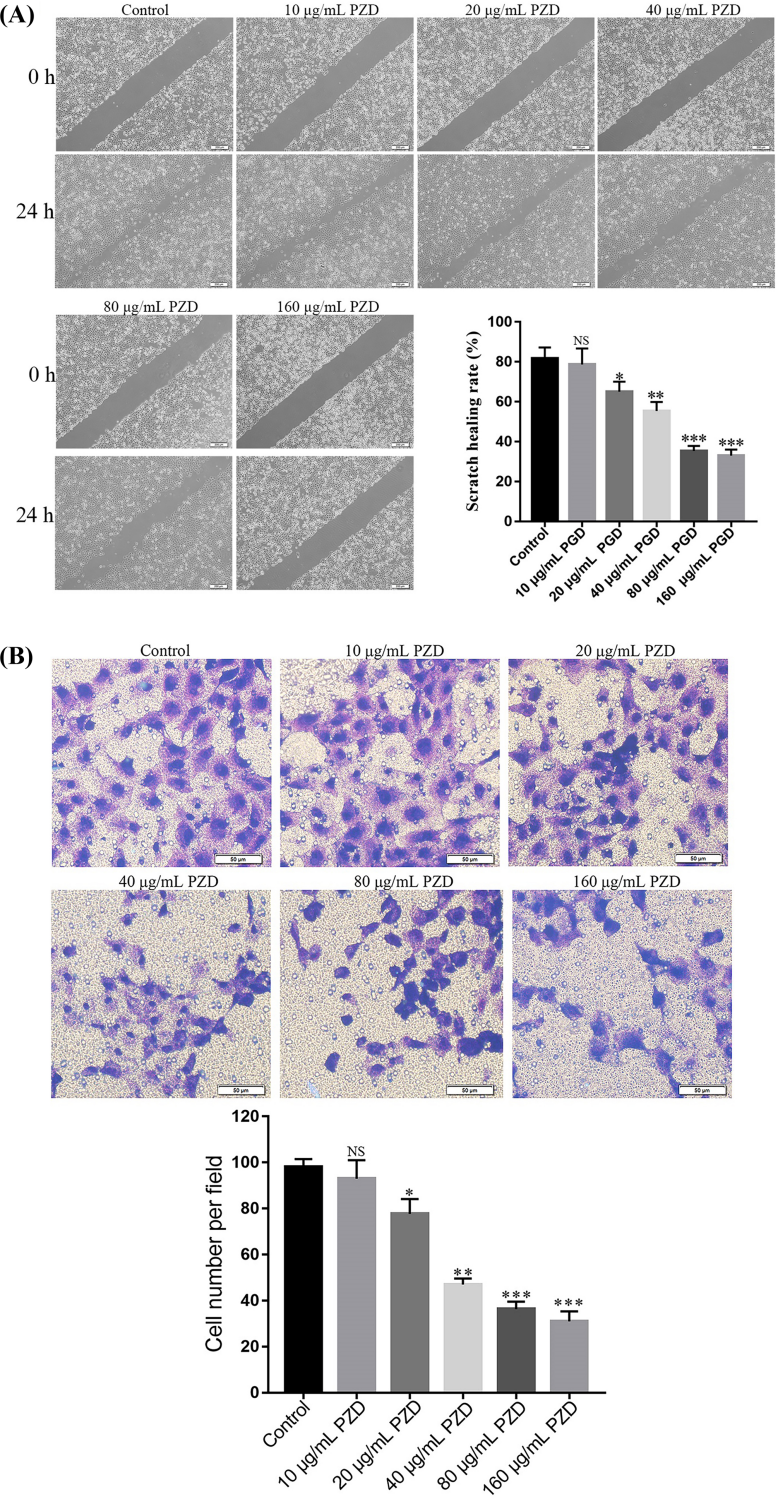
Effects of PZD extracts on the migration and invasion of H1299 cells. (**A**) Wound-healing assay results showed that PZD extracts significantly decreased cell migration in H1299 cells. (**B**) Transwell assay results showed that PZD extracts significantly decreased cell invasion in H1299 cells. * and ** represent *p* < 0.05 and *p* < 0.01 compared with the blank dose group, respectively.

To investigate the effects of PZD extract on the lung cancer cells *in vitro*, cell migration and invasion experiments were carried out. As shown in Fig. (**8A**), the wound-healing experiment indicated that the speed of migration was slower in NCI-H1299 cells treated with PZD extract, indicating that it could inhibit lung cancer cell migration at 80 and 160 μg/mL concentrations (*p* < 0.001). Moreover, compared with control groups, the speed of invasion was slower in NCI-H1299 cells treated with PZD extract (Fig. **8B**). These results indicated that the PZD extract could decrease cell migration and invasion *in vitro*.

### 3.9 PZD Extract Regulated the PI3K/AKT Signaling Pathway of NCI-H1299 Cells

Previous bioinformatics analysis showed that the regulatory target of the PZD components involved the AKT protein, so the WB experiment was used to verify whether PZD could regulate the malignant phenotypic characteristics of lung cancer cells through the PI3K/AKT signaling pathway. As shown in Fig. (**9**), PZD could significantly inhibit the expression of PI3K and AKT proteins at the concentration of 80 and 160 μg/mL and their phosphorylation level, indicating that PZD could regulate and inhibit the expression of PI3K/AKT signaling pathway.

## 4 DISCUSSION

PZD is a traditional Chinese medicinal decoction recorded by ancient physicians for relieving clinical diseases, such as cough and sputum. By searching the TCMSP database of traditional Chinese medicine decoction with the network pharmacology method, 16 kinds of effective active chemical components of *Pinellia* and *Zingiber officinale* were screened out, mainly including sterols, alkaloids, flavonoids, and cypress glycosides. The results of modern pharmacological research showed that these compounds play an important role in regulating tumorigenesis, suggesting that network pharmacology has important reference value for screening effective active ingredients of drugs [47-49]. The network of drugs-key chemical components-disease targets not only visualized the progress of the ancient Chinese medicine decoction but also suggested that pinasterol, β-sitosterol, carvendine, baicalein, coniferin, and other chemical components may play a key regulatory function in lung cancer targeted therapy [50]. β-Sitosterol has been found in many Chinese herbal medicines to significantly inhibit the proliferation of tumor cells, especially in lung cancer. β-sitosterol can target and regulate Trx/Trx1 reductase to induce apoptosis of lung cancer cells [51-55]. Furthermore, stigmasterol is also an important component of phytosterols, which is mainly isolated from soybeans and lentils. Studies have found that it has broad-spectrum anti-cancer, antibacterial, and anti-oxidant effects. It could be used to treat breast and colorectal cancer patients. Studies have shown that stigmasterol could significantly reduce the burden of metastatic tumors at cancer sites, mainly by reducing the continuous expression of pAKT, metastasis marker genes (alkaline phosphatase, matrix metalloproteinases, epithelial to mesenchymal transcription factors), vascular growth factor (vascular endothelial

growth factor), CD31, and continuous expression of cell proliferation antigen (Ki67, proliferative cell nuclear antigen) [56-59]. Baicalein has broad-spectrum physiological activities, such as antibacterial, antiviral, and anti-inflammatory, and it can exert antitumor effects through multiple targets and multiple pathways [60-64]. Moreover, carvendine has significant antitumor effects, along with anti-inflammatory, immune-regulating, and anti-viral effects. It mainly acts on potential targets, such as the efferent nervous system, ion channels, PDE10A, and coagulation factors [65]. At present, there are few studies on the anti-tumor effect of coniferin [66]. It has been found that its antioxidant and antibacterial activities were mainly achieved through scavenging 2,2-diphenyl-1-picrylhydrazyl (DPPH) free radicals [67]. This regulatory process has an important function in tumor cell apoptosis, implying that coniferin has a certain antitumor effect. In this study, it was found that PZD extract could inhibit the cell growth of lung cancer NCI-H1299 cells, but the multi-component coordination regulation mechanism of PZD was complex. Currently, single-use small-molecule drugs, such as clofoctol and ivacaftor, have been shown to inhibit the proliferation and progression of cancer stem cells by reducing the expression of *CD44*, *CD133*, and *Sox2* genes [68-70], but whether PZD inhibits the proliferation of lung cancer by regulating cancer stemness needs further research.

Furthermore, through the GO function enrichment analysis of the signal transduction pathway of disease target proteins, it was found that these proteins are mainly involved in drug response, signal transduction, positive regulation of cell proliferation, cell proliferation, cell apoptosis, and other biological function, such as synaptic transmission and positive regulation of cell migration. KEGG pathway analysis showed that the main pathways of these disease target genes involved serotonergic synapse, cGMP-PKG signaling pathway, adrenaline signaling in cardiomyocytes, calcium signaling pathway, p53 signaling pathway, platinum resistance, apoptosis, non-small cell lung cancer, bladder cancer, thyroid cancer, renal cell carcinoma, pancreatic cancer, and endometrial cancer. A large number of pathways have been shown to be closely related to tumor regulation and drug resistance pathways [71, 72], suggesting that PZD may treat lung cancer by regulating tumor cell signaling pathways.

Through the mutual mapping of drug target genes and disease target genes, combined with the PPI protein interaction network relationship for prediction, the results suggested that AKT1, TP53, FOS, MMP9, VEGFA, CASP3, and ADAR (1A, 1B, 2A, and 2C) may be the key core proteins for the treatment of lung cancer. AKT1 was found to be a key pathway target for antitumor activity, which is closely related to the prognosis and differentiation grade of lung cancer. A large number of studies on lung cancer mainly focused on AKT/mTOR/NF-κB, EGFR-AKT-mTOR, and PI3K/ AKT/mTOR signal pathways. These pathways were found to be closely related to the proliferation, invasion, and migration of tumor cells [73-75]. Interestingly, our experimental results proved that PZD could inhibit the phosphorylation level of PI3K and AKT protein activation, indicating that PZD could inhibit the proliferation, migration, and invasion of lung cancer cells by regulating the PI3K/AKT signaling pathway.

In conclusion, based on the research method of network pharmacology, this study explored the key active compounds and target proteins of *Pinellia-Zingiber officinale* and their relationship with lung cancer regulatory pathways, indicating that PZD could regulate lung cancer by multi-component, multi-target, multi-channel synergistic regulation mechanism. This paves the way for the theoretical foundation for deeper experimental investigation and provides new insights into its specific molecular regulation mechanisms for future studies.

## CONCLUSION

The pharmacological mechanism of PZD inhibiting lung cancer cells was explored by combining network pharmacology prediction and experimental verification. We demonstrated that PZD may mainly inhibit the proliferation, migration, and invasion of lung cancer cells by regulating the PI3K/AKT signaling pathways. Our research further suggested that a combination of network pharmacology prediction and experimental validation may provide useful tools for the description of the mechanism of action of traditional Chinese medicine. The potential therapeutic effects of PZD on lung cancer cells may benefit from further clinical trials of lung cancer patients treated with PZD.

## AUTHORS’ CONTRIBUTIONS

Revision for important intellectual content was carried out by FF, PH, and LP. Preparation of the manuscript was done by PH, LP, JC, and XKT under the supervision of FF.

## Figures and Tables

**Fig. (1):**
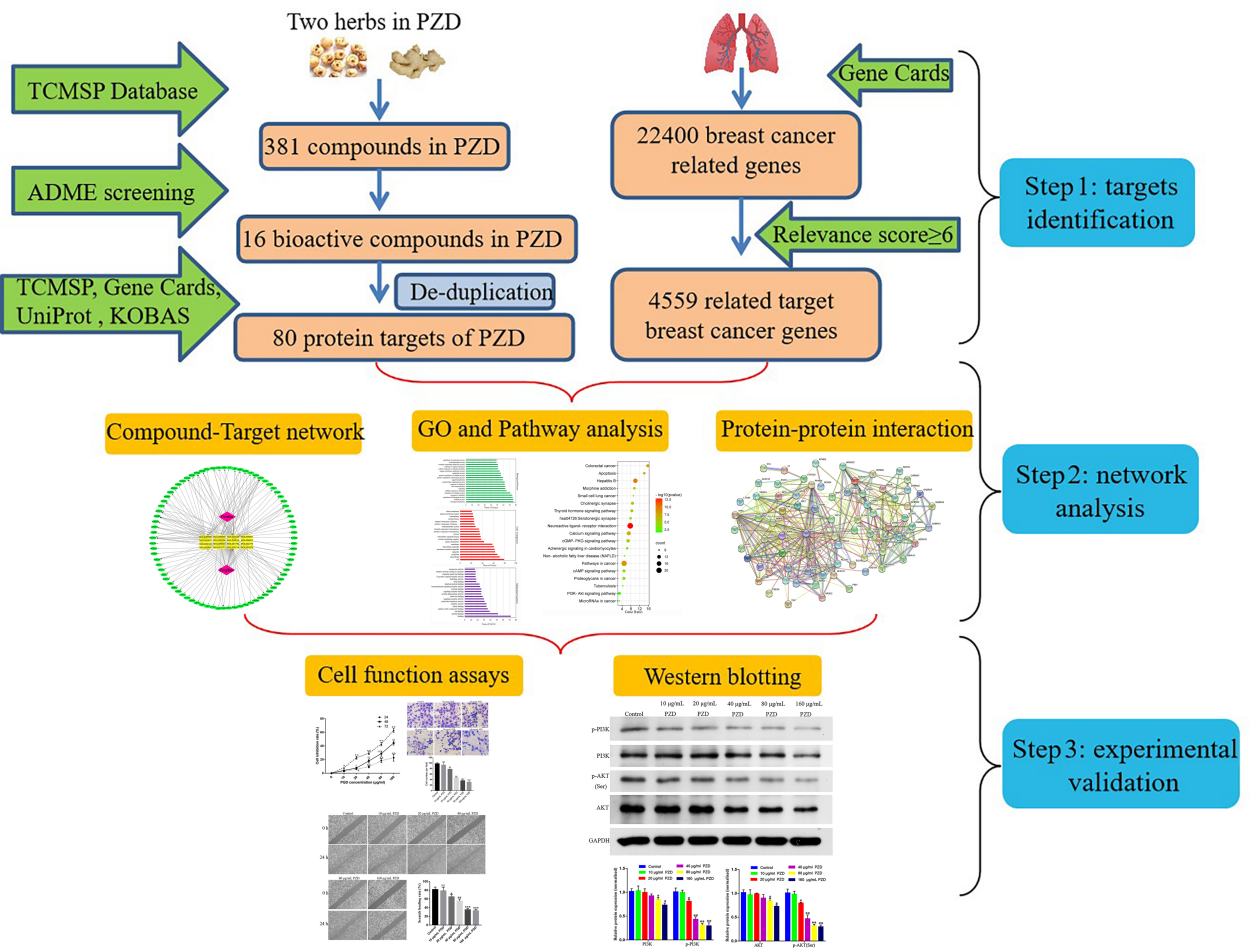
The technical strategy of the current study.

**Fig. (2):**
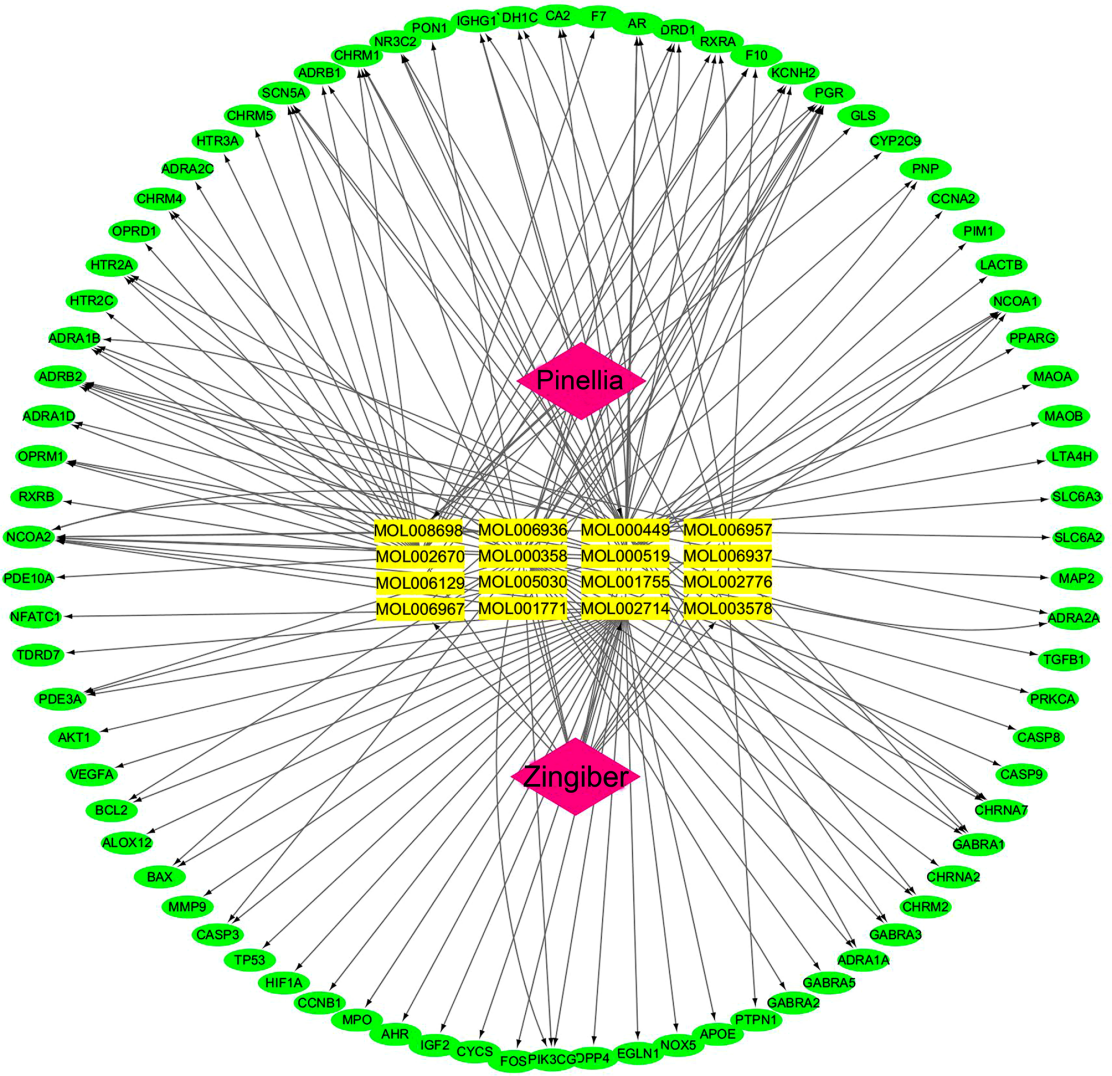
The drugs-bioactive components-disease targets network for PZD on lung cancer. The purple nodes represent *Pinellia ternata*-*Zingiber officinale* drugs, the yellow nodes represent candidate active compounds, and the green nodes represent potential protein targets. The edges represent the interactions between these nodes.

**Fig. (4):**
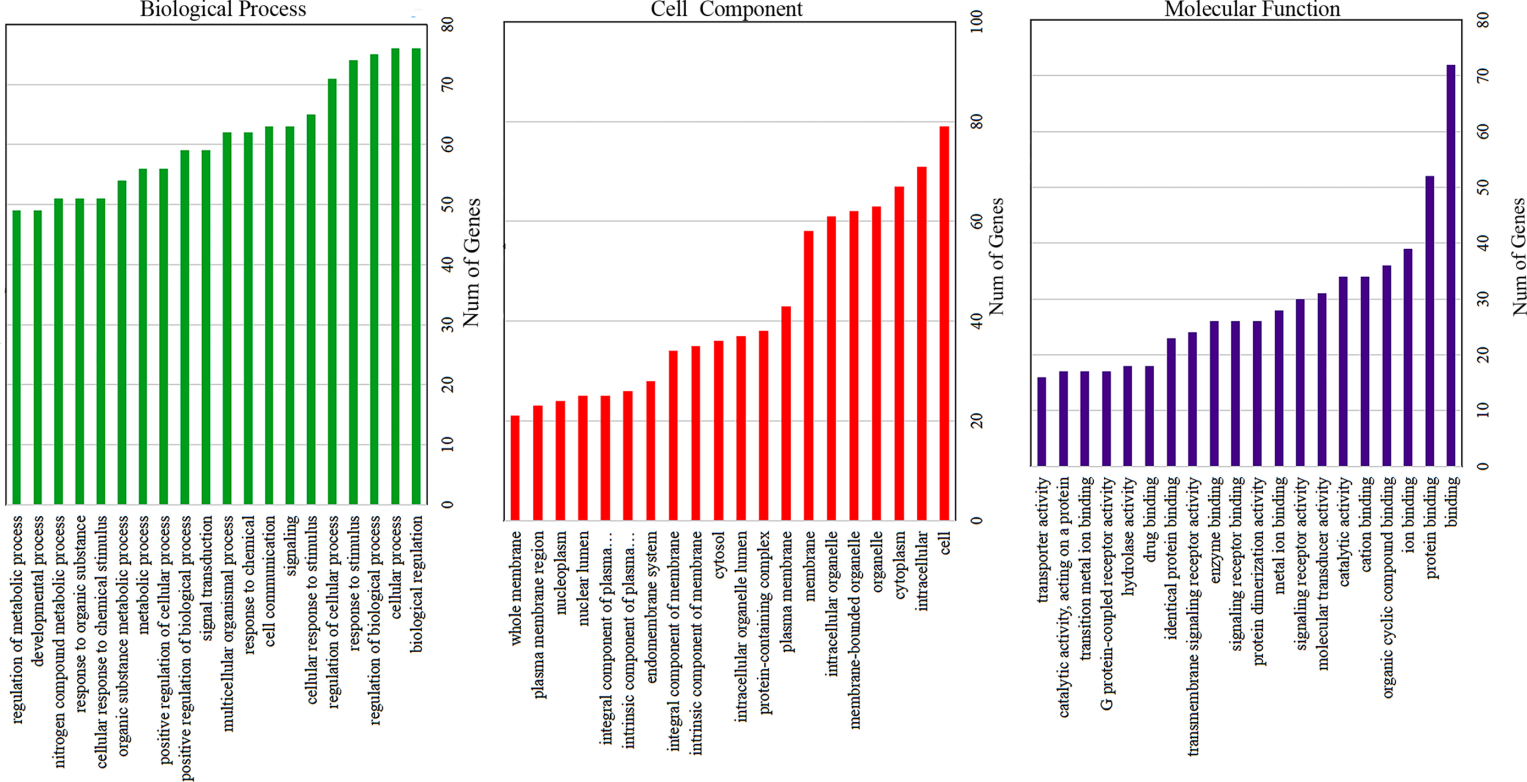
GO Enrichment analysis of protein targets of PZD bioactive compounds in lung cancer.

**Fig. (5):**
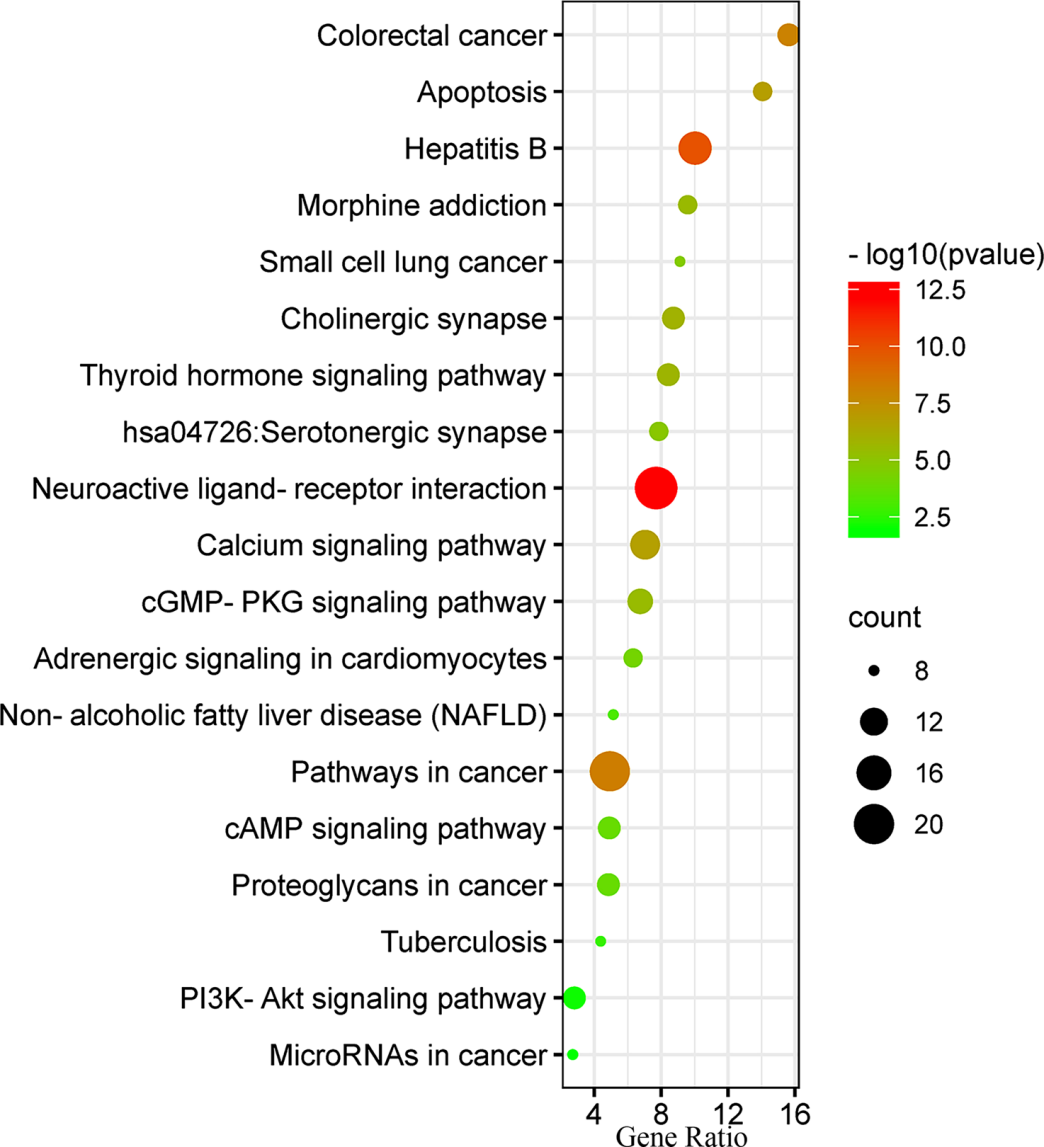
KEGG pathway analysis of protein targets of PZD bioactive compounds in lung cancer.

**Fig. (9):**
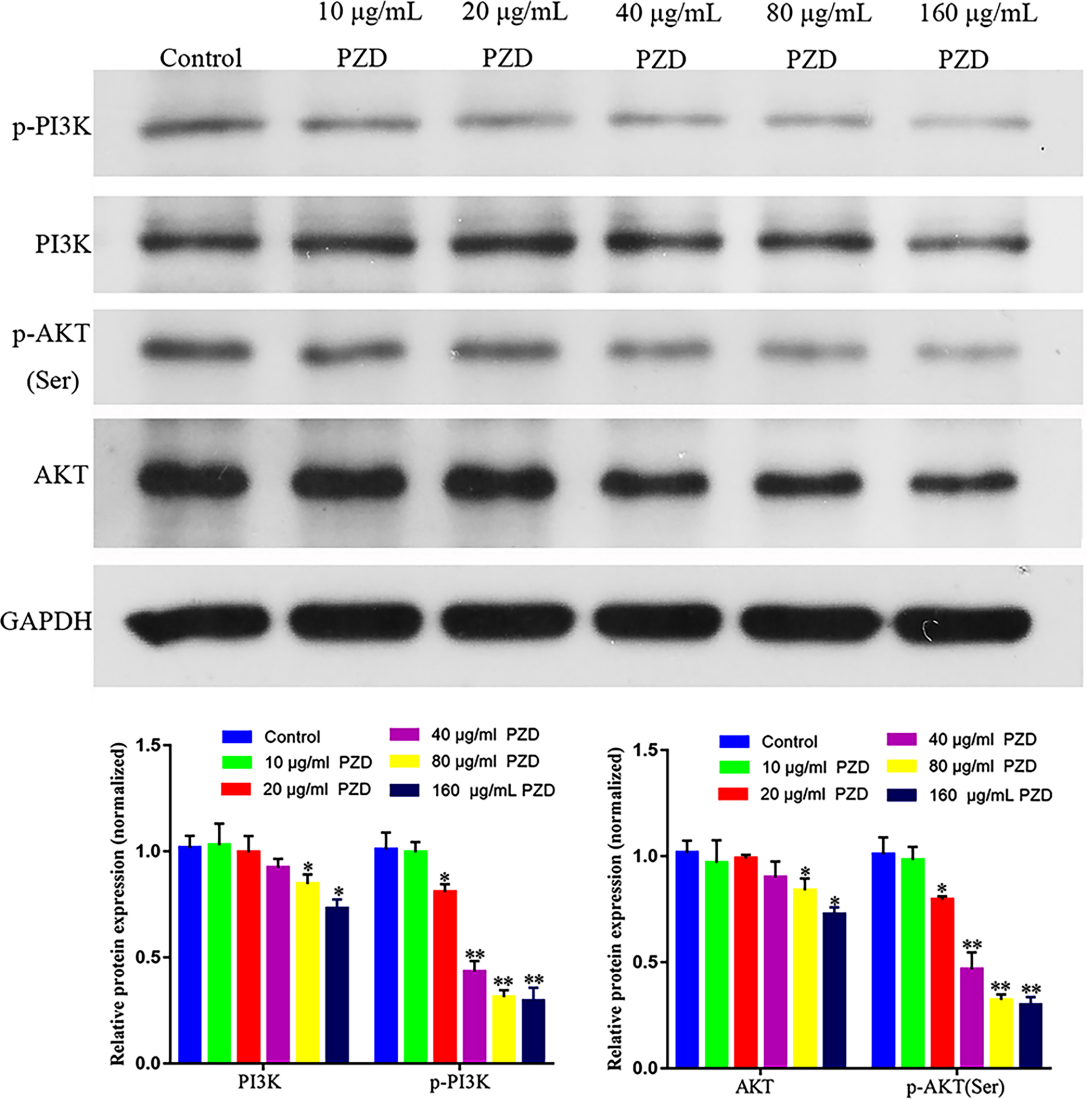
PZD extract regulated the PI3K/AKT signaling pathway of NCI-H1299 cells. Western blot experiments showed that PZD could significantly inhibit the expression of PI3K and AKT protein at the concentrations of 80 and 160 μg/mL and inhibit their phosphorylation level. * and ** represent *p* < 0.05 and *p* < 0.01 compared with the blank dose group, respectively.

**Table 1:** Basic information of 16 bio-active compounds in PZD.

**Number**	**Molecular Name**	**OB (%)**	**DL**	**Molecules Structure**	**Herb**
MOL1755	24-Ethylcholest-4-en-3-one	36.08	0.76	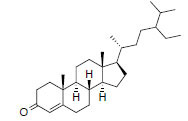	*Pinellia*
MOL449	Stigmasterol	43.83	0.76	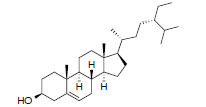	*Pinellia* *Zingiber*
MOL358	Beta-sitosterol	36.91	0.75	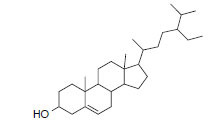	*Pinellia* *Zingiber*
MOL5030	Gondoic acid	30.70	0.20	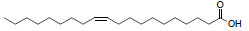	*Pinellia*
MOL1771	Poriferast-5-en-3beta-ol	36.91	0.75	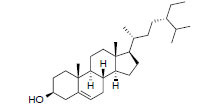	*Zingiber*
MOL2670	Cavidine	35.64	0.81	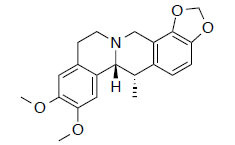	*Pinellia*
MOL2776	Baicalin	40.12	0.75	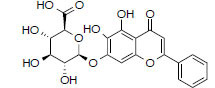	*Pinellia*
MOL6129	6-methylginge- diacetate2	48.73	0.32	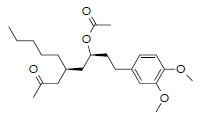	*Zingiber*
MOL2714	Baicalein	33.52	0.21	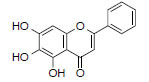	*Pinellia*
MOL519	Coniferin	31.11	0.32	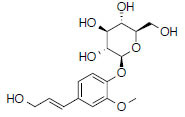	*Pinellia*
MOL6936	10,13-eicosadienoic	39.99	0.20	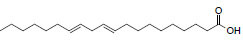	*Pinellia*
MOL3578	Cycloartenol	38.69	0.78	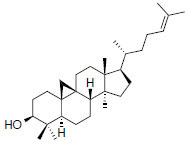	*Pinellia*
MOL695	(3S,6S)-3-(benzyl)-6-(4-hydroxybenzyl)piperazine-2,5-quinone	46.89	0.27	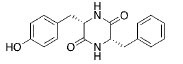	*Pinellia*
MOL6937	12,13-epoxy-9-hydroxynonadeca-7,10-dienoic acid	42.15	0.24	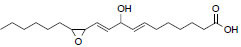	*Pinellia*
MOL6967	Beta-D-Ribofuranoside	44.72	0.21	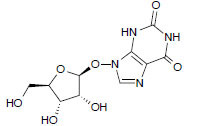	*Pinellia*
MOL8698	Dihydrocapsaicin	47.17	0.19	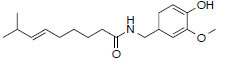	*Zingiber*

**Table 2:** Binding free energy for key chemical components to target protein.

**Chemical Components**	**Target Protein**	**Binding Free Energy/kcal·mol^-1^**
Cavidine	VEGFA	-9.7
Baicalin	MMP9	-9.0
Stigmasterol	AKT1	-8.1
Coniferin	FOS	-6.8
Beta-sitosterol	ADAR1	-6.3

## Data Availability

The authors confirm that the data supporting the findings of this study are available within the article.

## LIST OF ABBREVIATIONS

ADME: Absorption, Distribution, Metabolism, Excretion
GO: Gene Ontology
KEGG: Kyoto Encyclopedia of Genes and Genomes
PPI: Protein-protein Interaction
PZD: *Pinellia ternata-Zingiber officinale* Decoction
TCM: Traditional Chinese Medicine
